# Renal Functional Response-Association With Birth Weight and Kidney Volume

**DOI:** 10.1016/j.ekir.2023.02.1079

**Published:** 2023-02-20

**Authors:** Bjørn Steinar Lillås, Camilla Tøndel, Toralf Melsom, Bjørn Odvar Eriksen, Hans-Peter Marti, Bjørn Egil Vikse

**Affiliations:** 1 Department of Medicine, Haugesund Hospital, Haugesund, Norway; 2 Department of Clinical Medicine, University of Bergen, Bergen, Norway; 3 Department of Pediatrics, Haukeland University Hospital, Bergen, Norway; 4 Metabolic and Renal Research group, UiT The Arctic University of Norway, Tromsø, Norway; 5 Section of Nephrology, University Hospital of North Norway, Tromsø, Norway; 6 Department of Medicine, Haukeland University Hospital, Bergen, Norway

**Keywords:** iohexol clearance, low birth weight, renal functional response, renal stress test, renal functional reserve, kidney volume, measured GFR

## Abstract

**Introduction:**

Renal functional response (RFR) is the acute increase in glomerular filtration rate (GFR) after a protein load. Low RFR is a marker of single nephron hyperfiltration. Low birth weight (LBW) is associated with reduced number of nephrons, lower kidney function, and smaller kidneys in adults. In the present study, we investigate the associations among LBW, kidney volume, and RFR.

**Methods:**

We studied adults aged 41 to 52 years born with either LBW (≤2300 g) or normal birth weight (NBW; 3500–4000 g). GFR was measured using plasma clearance of iohexol. A stimulated GFR (sGFR) was measured on a separate day after a protein load of 100 g using a commercially available protein powder, and RFR was calculated as delta GFR. Kidney volume was estimated from magnetic resonance imaging (MRI) images using the ellipsoid formula.

**Results:**

A total of 57 women and 48 men participated. The baseline mean ± SD GFR was 118 ± 17 ml/min for men and 98 ± 19 ml/min for women. The overall mean RFR was 8.2 ± 7.4 ml/min, with mean RFR of 8.3 ± 8.0 ml/min and 8.1 ± 6.9 ml/min in men and women, respectively (*P* = 0.5). No birth-related variables were associated with RFR. Larger kidney volume was associated with higher RFR, 1.9 ml/min per SD higher kidney volume (*P* = 0.009). Higher GFR per kidney volume was associated with a lower RFR, −3.3ml/min per SD (*P* < 0.001).

**Conclusion:**

Larger kidney size and lower GFR per kidney volume were associated with higher RFR. Birth weight was not shown to associate with RFR in mainly healthy middle-aged men and women.

## Graphical abstract

**Figure:**
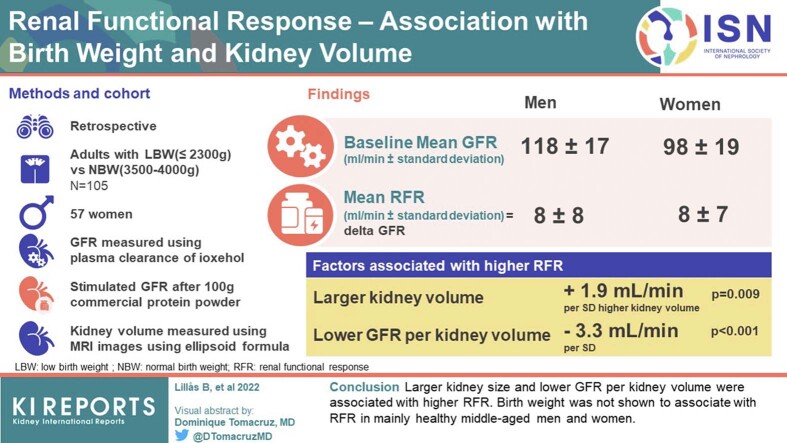

RFR, also known as renal functional reserve, measures the kidneys’ ability to increase their function in response to various external demands.^1^, ^2^, ^3^ The presence of such a response can be measured using a renal stress test, usually by an oral load of protein.^4^^,^^5^ Such a protein load results in an acute rise in GFR and we can thus measure a sGFR. By subtracting the baseline GFR (bGFR) from the sGFR, the RFR can be calculated. RFR is given either as the absolute delta GFR or as a percentage of the bGFR. The significance of RFR is debatable,^1^^,^^6^ but diminished RFR has been suggested as a predictor of future GFR loss^7^^,^^8^ and a marker of single nephron hyperfiltration.^9^

The impact of birth weight on adult kidney health is well documented in previous studies.^10^^,^^11^ It has been shown that LBW is associated with lower nephron number, albuminuria, hypertension, lower kidney function, and increased risk of kidney failure.^12^, ^13^, ^14^ We have previously shown in a mainly healthy cohort that women aged 40 to 50 years who were born with LBW have lower measured GFR (mGFR)^15^ and lower kidney volume^16^ than women born with NBW. In the male cohort of the study, no such difference was found. A possible hypothesis could be that the men with LBW were using more of their renal capacity, which could be demonstrated by diminished RFR.

In the participants of the previous study,^15^ we measured RFR after an oral load of 100 g of protein.^17^ This study explores how RFR in an adult cohort associates with birth weight and kidney volume in women and men. We hypothesized a positive association between birth weight and RFR and that sex differences in RFR could explain the previous findings of sex differences in the association between birth weight and kidney volume and function. We also hypothesized a positive association between adult kidney volume and RFR.

## Methods

### Study Population

The study population was part of a previously described cohort study^15^ comparing presumably healthy adults aged 41 to 52 years born either with LBW (≤ 2300 g) or NBW (3500–4000 g). An invitation to participate in the study was sent from the Medical Birth Registry of Norway. Participants were randomly selected using the following inclusion criteria: born in Norway between 1967 and 1976; currently residing in the area surrounding Haugesund, Norway; singleton birth; and birth weight either <2300 g or between 3500 and 4000 g. Exclusion criteria were treatment for hypertension, diabetes mellitus, eGFR <60 ml/min per 1.73 m^2^, and cancer within the last 5 years. The Medical Birth Registry of Norway is a nationwide registry with compulsory registration of all births and pregnancies terminated after the sixteenth week of gestation.^18^ Data are available from 1967. The Medical Birth Registry of Norway provided data on birth weight, gestational age, birth weight for gestational age, maternal preeclampsia, and body length at birth.

### Overview of the Study

Participation in the study required attendance on 3 separate days. On the first day, we measured bGFR, on the second day we measured sGFR, and on the third day, we measured kidney volume using MRI. For the first and second days, the tests were performed in the morning with the participants fasting, whereas the MRI was done in the afternoon while the participants were not fasting. The 3 days were separated by at least 1 week, except for 1 participant who underwent the MRI study on the same afternoon as the sGFR.

bGFR was measured using plasma clearance of iohexol after a single injection of 5 ml of 300 mg I/ml iohexol (Omnipaque, Oslo, Norway). Blood samples were collected after 2 and 4 hours, and calculations were done according to the method of Jødal and Brøchner-Mortensen.^19^

sGFR was measured using the same method for plasma clearance of iohexol. However, in the stimulated test, the participants ingested a protein load in the form of a commercially available protein powder (“TriWhey” from MyoNutrition; Melhus, Norway) mixed according to the manufacturer. The total amount of protein ingested was 100 g and we used a fixed amount for all participants, regardless of weight and sex. This was an adaptation of the original protein stimulation test by Bosch,^2^ and the method using iohexol clearance has been described in a previous paper.^17^ To ensure complete washout of iohexol, the sGFR was measured at least 1 week after the bGFR test.

The RFR was calculated after the stress test as delta GFR, using the formula: RFR = sGFR − bGFR. We used non-body surface areas (non-BSAs) corrected mGFR (both baseline and stimulated) in the calculation of RFR as well as for adjustment in the regression models unless otherwise specified.

Kidney volume was calculated from MRI images using the ellipsoid formula:

Volume = $\frac{\pi }{6}*length*width*depth$. A complete description of the kidney volume calculation is given in a previous paper.^16^ The kidney utilization ratio was defined and calculated as the mGFR (without BSA correction) divided by the total kidney volume. Assuming an equal distribution of nephrons and ignoring variations in kidney volume without nephrons, we suggest this ratio is proportional to the mean single nephron GFR.

Height and weight were measured on the first day of participation and rounded to the nearest cm and kg, respectively. Body mass index (BMI) was calculated as weight/height^2^ (kg/m^2^), and BSA (m^2^) was calculated according to the method of DuBois.^20^

Blood pressure was measured seated 3 times, once immediately before injection of iohexol and then at 15 and 30 minutes after the injection. We used the mean of the 2 latter measurements from the first day for analysis.

All participants provided a midstream urine sample in the morning on 3 consecutive days, and the albumin creatinine ratio was calculated. From the 3 samples, we used the median value per participant for analysis.

### Statistics

We used R studio version 4.2.1 (R Foundation for Statistical Computing, Vienna, Austria), for all statistics.^21^ Normally distributed data are presented as mean ± SD and non-normally distributed data as median (min, max). We chose a significance level of 0.05 for all tests. Because the size of the study sample was determined from an *a priori* power calculation using differences in mGFR,^15^ we performed a *post hoc* power test for independent t-test of unequal sample sizes based on estimates from our pilot study.^17^ Using a power of 80%, significance level of 0.05, and the actual sample sizes of this study, we would be able to find a difference in mean RFR of at least 3.5 ml/min, which was deemed sufficient to find a clinically significant difference. We compared birth-related variables, body composition, blood pressure, and RFR between birth weight groups and sex using a 2-way analysis of variance with a type III sum of squares because of unequal group sizes. A linear regression model was fitted using RFR as the dependent variable. We used various birth-related variables, age, body composition, blood pressure, and kidney function as independent variables. We fitted 3 models as follows: (i) model 0 was each variable unadjusted; (ii) model 1, adjusted for sex, age, height, and weight; and (iii) model 2, adjusted for sex, age, height, weight, and bGFR. Similarly, the same variables were tested for interaction with the birth weight group. In the regression analysis, the estimates of continuous variables are shown per SD. This was to allow for a better comparison of their individual effects. All regression analyses were performed both for the total cohort as well as sex-stratified. We performed a sensitivity analysis where the dependent variable was changed to RFR in percentage and RFR using BSA corrected GFR. A mediation analysis was performed using the R-package “mediation” (version 4.5.0) with the percentile method using 5000 simulations.

## Results

The Medical Birth Registry of Norway invited 400 persons and 176 responded (44%). After receiving more information about the project, 29 responders withdrew for personal reasons. Another 42 responders were excluded because of hypertension (14); medications (8); diabetes (4); and other reasons (16), including among others, cancer and allergies. We included a total of 105 study participants of which 57 (including 23 males) were included in the LBW group and 48 (including 24 males) in the NBW group. We had complete study data on 101 of 105 participants. One participant had missing gestational age, 2 participants had missing kidney volume, and 1 participant had both missing gestational age and kidney volume. The birth weight groups were similar in age at examination and body composition, except the LBW group being shorter than the NBW group (170 cm vs. 173 cm, *P* = 0.04; Table 1). With a mean BMI of 26.6 kg/m^2^, the whole sample was slightly overweight; however, we could not find any significant difference in BMI between the birth weight groups, nor between males and females. As previously reported, the female LBW group had lower mGFR^15^ (*P* = 0.006) and smaller kidneys^16^ (*P* = 0.002) than the female NBW group; in men, no such difference was seen. The mean kidney utilization ratio (GFR per kidney volume) was 0.355 ± 0.052 ml/min/ml with similar values for both birth weight groups as well as both men and women. Median albumin-to-creatinine ratio was 0.4 mg/mmol. The value of the only participant with a median albumin-to-creatinine ratio >3.0 mg/mmol was 3.2 mg/mmol. A complete description of the participants stratified by birth weight group and sex is shown in Table 1.

**Table 1:** Characteristics of participants at birth and examination

Characteristics	Male LBW	Male NBW	Female LBW	Female NBW	*P* BW group	*P* sex
Number, n	23	24	34	24		
Birth weight-g	1983 ± 260	3745 ± 120	1919 ± 314	3736 ± 136	<0.001	0.4
Birth weight per gestational age-SD	−0.86 (−3.88, 1.78)	0.085 (−0.42, 1.42)	−1.33 (−4.65, 1.07)	0.37 (−0.28, 1.5)	<0.001	0.8
Gestational age-wks	34 ± 3	40 ± 2	35 ± 3	40 ± 1	<0.001	0.4
Age at examination-yrs	47 ± 3	47 ± 2	47 ± 3	46 ± 3	0.4	0.5
Weight at examination-kg	87.4 ± 18.9	83.8 ± 10.1	71.2 ± 14.9	74.1 ± 15.6	0.9	<0.001
Height at examination-cm	177 ± 7	179 ± 5	165 ± 4	167 ± 6	0.04	<0.001
Body mass index	27.8 ± 5.6	26.1 ± 3	26.2 ± 5.3	26.4 ± 5.4	0.5	0.5
Body surface area	2.04 ± 0.20	2.02 ± 0.12	1.77 ± 0.17	1.83 ± 0.18	0.5	<0.001
Systolic blood pressure-mm Hg	131 ± 19	120 ± 8	123 ± 16	118 ± 14	0.01	0.07
Diastolic blood pressure-mm Hg	81 ± 12	72 ± 8	72 ± 11	67 ± 9	0.001	0.001
Measured GFR-ml/min	119 ± 19	117 ± 14	93 ± 15	106 ± 21	0.07	<0.001
Measured GFR-ml/min/1.73 m^2^	101 ± 15	100 ± 11	90 ± 12	101 ± 14	0.06	0.03
Total kidney volume-ml	340 ± 65	347 ± 51	258 ± 48	302 ± 51	0.01	<0.001
GFR per kidney volume	0.357 ± 0.060	0.341 ± 0.041	0.364 ± 0.046	0.356 ± 0.063	0.3	0.3
GFR per body weight	1.398 ± 0.245	1.404 ± 0.166	1.332 ± 0.249	1.457 ± 0.217	0.1	0.8
Urinary albumin/creatinine ratio-mg/mmol	0.4 (0.0, 3.2)	0.4 (0.0, 2.6)	0.3 (0.0, 1.6)	0.3 (0.0, 0.9)	0.7	0.3
RFR-ml/min	8.9 ± 8.86	7.8 ± 7.21	8.3 ± 7.25	7.9 ± 6.46	0.6	0.9
RFR above 0	18 (78 %)	22 (92 %)	31 (91 %)	20 (83 %)	0.8	0.7
RFR above 5	16 (70 %)	16 (67 %)	21 (62 %)	17 (71 %)	0.7	0.8
RFR above 7.5	12 (52 %)	12 (50 %)	17 (50 %)	12 (50 %)	0.9	0.9
RFR above 10	10 (43 %)	7 (29 %)	14 (41 %)	10 (42 %)	0.5	0.6
RFR above 15	5 (22 %)	1 (4 %)	7 (21 %)	3 (12 %)	0.08	0.6

GFR, glomerular filtration rate; LBW, low birth weight; NBW, normal birth weight; RFR, renal functional response.

Description of participants by birth weight group and sex. Group differences tested by 2-way analysis of variance and *P* - value shown.

**Figure 1:**
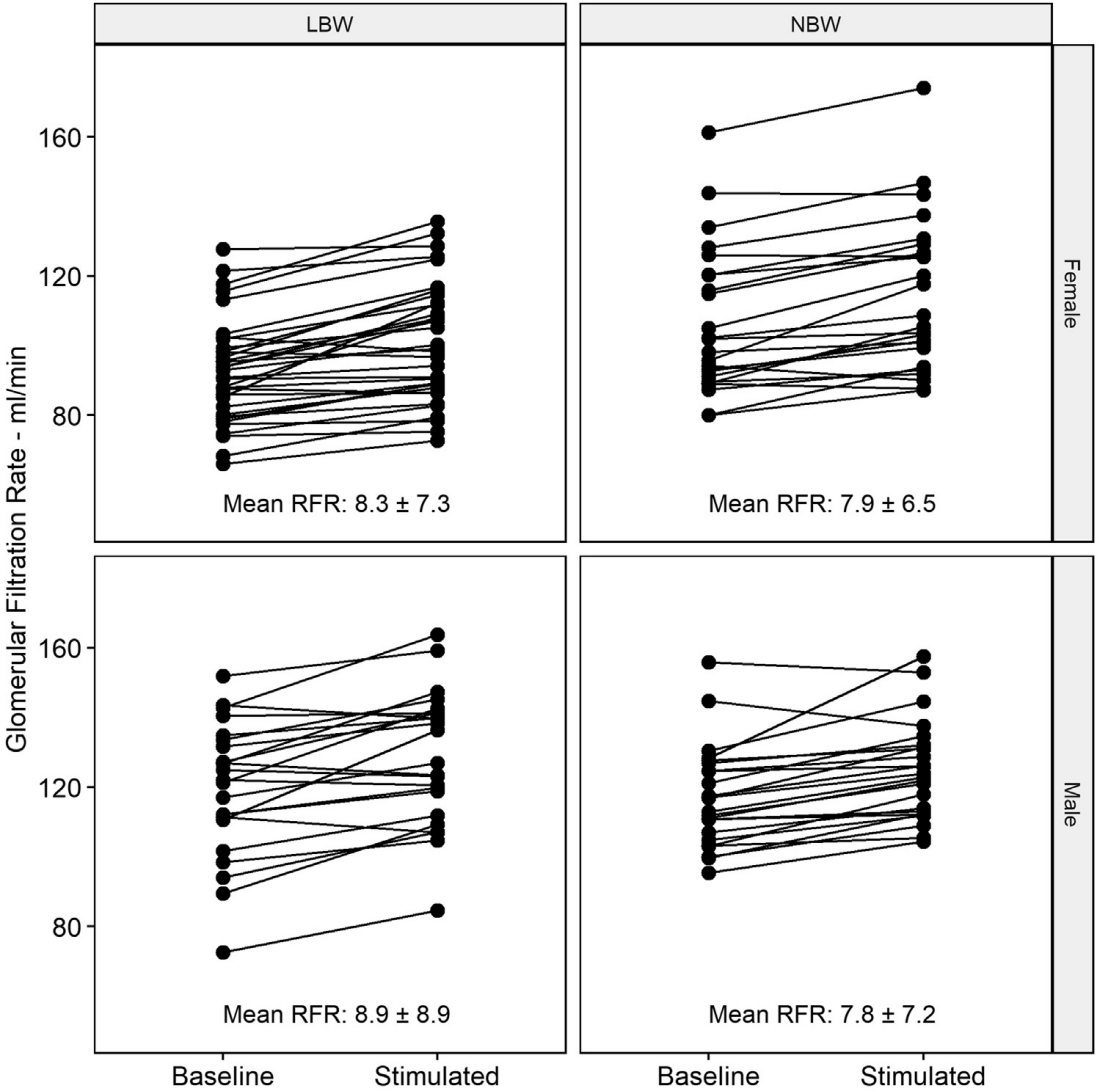
Changes in GFR after protein load. Individual changes from baseline GFR after stimulation with 100 g protein in the form of a protein shake. Results are stratified by birth weight group and by sex. LBW = Low birth weight (<2300 g) and NBW = Normal birth weight (3500–4000 g). Mean RFR ± SD in % given for each group. LBW, low birth weight; NBW, normal birth weight; RFR, renal functional response.

After the protein stimulation, mGFR increased in 91 participants (87%) as compared with baseline mGFR. The individual change from baseline to sGFR is shown in Figure 1. Mean RFR was 8.2 ± 7.4 ml/min with a minimum value of −7.2 ml/min and a maximum of 29.2 ml/min. We could not find any significant difference in RFR between the birth weight groups, nor between the sexes (Table 1; the distribution is shown in Supplementary Figure S1). Because RFR was not normally distributed, we did a separate test using Mann-Whitney U with similar nonsignificant results (not shown). Fourteen participants (13%) had negative RFR. This was similar in both birth weight groups and both sexes (*P* = 0.8 and 0.7, respectively). Other cutoff points were tested and showed no significant pattern between birth weight groups or between men and women (Table 1).

**Figure 2:**
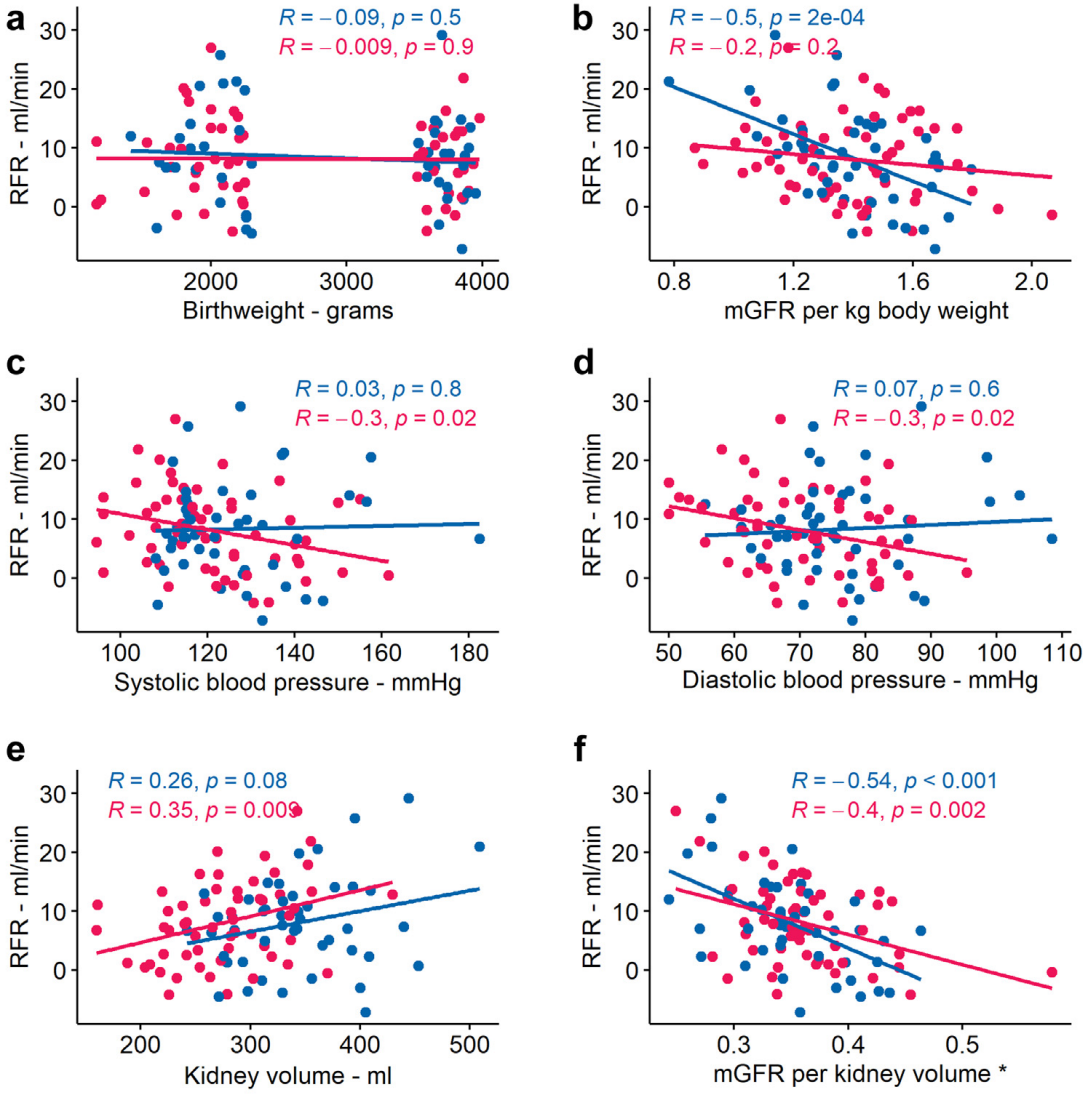
Association between RFR and various variables using regression Legend: Blue dots, lines, and text represent male participants; pink dots, lines, and text represent female participants. For all plots, dependent variable is RFR (ml/min). The independent variables are as follows: (a) birth weight in grams, (b) mGFR per kg body weight in ml/min/kg, (c) systolic blood pressure in mmHg, (d) diastolic blood pressure in mm Hg, (e) kidney volume in ml, and (f) measured GFR per kidney volume in ml/min/ml. mGFR, measured GFR; RFR, renal functional response.

The associations between RFR and other variables in the total cohort, including tests for interactions between each variable and sex are shown in Table 2. There were no significant interactions between the birth weight group and any of the variables in Table 2 (not shown). Sex-stratified analyses are shown in Supplementary Table S1. We found no association between any of the birth-related variables and RFR, and this was similar both for the whole cohort and in the sex-stratified analysis (see Table 2, Figure 2a, and Supplementary Table S1). In a sensitivity analysis, we found that this association was not affected by the method of estimating RFR (Supplementary Table S2). Age was significantly negatively associated with RFR, with similar results for men and women. Body composition (weight, height, BMI, and BSA) was associated with RFR. However, these associations showed varying estimates, directions, and significance among the 3 main models and between men and women. Weight showed a positive association with RFR (estimate per SD 1.63, *P* = 0.02). Although the sex interaction was nonsignificant, in the sex-stratified analysis, this association was only positive for men (see Supplementary Table S1). In the sensitivity analysis with varying methods of RFR estimation, we found that the effect of body composition on RFR was lost when RFR was measured in percent or corrected for BSA (see Supplementary Table S2). Systolic blood pressure, but not diastolic blood pressure, was associated with lower RFR after adjusting for age, sex, height, and weight (Figure 2c and d). Kidney volume was positively associated with RFR (Table 2 and Figure 2e). GFR per kidney volume was negatively associated with RFR with similar estimates and significance in all 3 models and this was not affected by sensitivity analysis (Table 2, Figure 2f, and Supplementary Table S2). GFR per body weight was also significantly negatively associated with RFR, except in model 2. In the sex-stratified analysis, this was only seen in men, whereas the sex interaction was nonsignificant. However, when analyzing only GFR per body weight and its sex-interaction, this was significant (*P* = 0.01, not shown). The association between baseline GFR and RFR is shown with a Bland Altman plot in Supplementary Figure S2.

**Table 2:** Association between renal functional response and various variables using linear regression

Characteristics	Model 0			Model 1			Model 2			Interaction
	Estimate	95% CI	*P*-value	Estimate	95% CI	*P*-value	Estimate	95% CI	*P*-value	*P*-value
Low birth weight	0.71	(−2.16, 3.58)	0.6	0.94	(−1.94, 3.82)	0.5	0.16	(−2.66, 2.97)	0.9	0.8
Birth weight	−0.34	(−1.78, 1.10)	0.6	−0.5	(−1.96, 0.96)	0.5	−0.03	(−1.47, 1.42)	0.9	0.6
Birth weight per gestational age	0.33	(−1.11, 1.76)	0.7	0.04	(−1.41, 1.49)	0.9	0.48	(−0.94, 1.90)	0.5	0.4
Gestational age	−0.42	(−1.86, 1.01)	0.6	−0.43	(−1.87, 1.01)	0.6	−0.16	(−1.56, 1.24)	0.8	0.8
Preterm	1.05	(−1.90, 3.99)	0.5	0.94	(−2.05, 3.94)	0.5	0.65	(−2.24, 3.53)	0.7	0.8
Age	−1.15	(−2.57, 0.27)	0.1	−1.47	(−2.88, −0.06)	0.04	−1.65	(−3.01, −0.29)	0.02	0.8
Weight	1.63	(0.23, 3.03)	0.02	2.23	(0.63, 3.84)	0.007	3.62	(1.83, 5.41)	<0.001	0.5
Height	0.25	(−1.19, 1.68)	0.7	−0.36	(−2.50, 1.79)	0.7	0.06	(−2.02, 2.14)	0.9	0.04
Body mass index	1.68	(0.28, 3.08)	0.02	−4.04	(−19.15, 11.06)	0.6	−3.26	(−17.79, 11.27)	0.7	0.06
Body Surface Area	1.31	(−0.11, 2.73)	0.07	−10.98	(−34.18, 12.21)	0.3	−4.97	(−27.71, 17.76)	0.7	0.9
Systolic blood pressure	−0.99	(−2.42, 0.43)	0.2	−2.3	(−3.90, −0.69)	0.006	−1.9	(−3.49, −0.31)	0.02	0.1
Diastolic blood pressure	−0.82	(−2.25, 0.61)	0.3	−1.46	(−3.05, 0.13)	0.07	−0.97	(−2.56, 0.61)	0.2	0.06
Kidney volume	1.87	(0.47, 3.27)	0.009	2.43	(0.46, 4.39)	0.02	5.11	(3.13, 7.09)	<0.001	0.3
Measured GFR	−0.56	(−2.00, 0.87)	0.4	−2.82	(−4.66, −0.99)	0.003	NA	NA	NA	0.4
GFR per kidney volume	−3.34	(−4.62, 2.07)	<0.001	−3.46	(−4.71, −2.22)	<0.001	−3.13	(−4.50, −1.77)	<0.001	0.5
GFR per kg body weight	−2.31	(−3.67, −0.94)	0.001	−2.16	(−3.77, −0.55)	0.009	1.09	(−3.46, 5.65)	0.6	0.1

CI, confidence interval; GFR, glomerular filtration rate.

All estimates are given per SD, except the dichotome variables low birth weight and preterm. Model 0 is unadjusted, Model 1 is adjusted for sex, age, height and weight, and Model 2 is adjusted for sex, age, height, weight, and absolute GFR. Interaction is the p-value for the interaction between the main variable and sex in Model 2.

**Figure 3:**
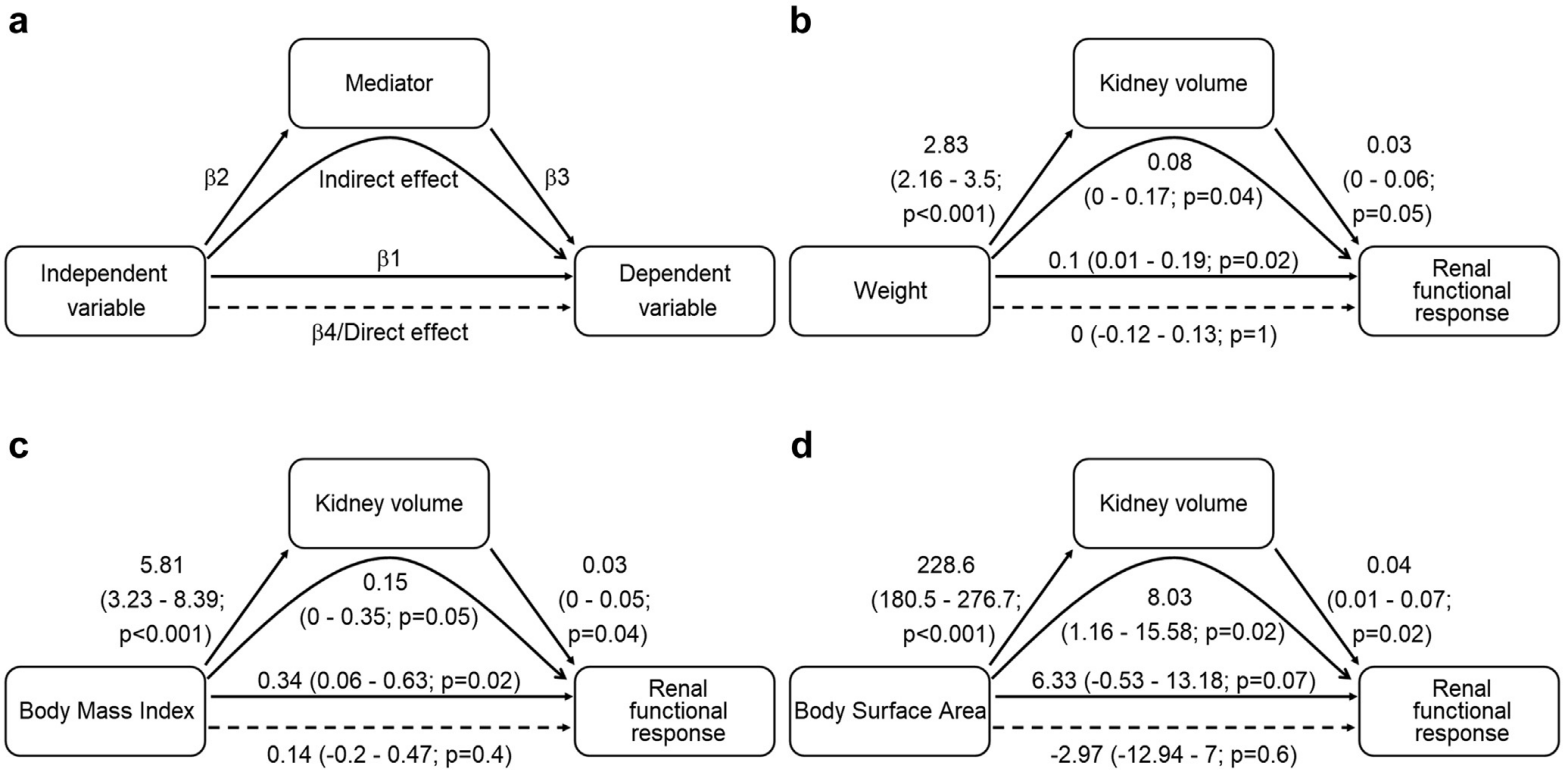
Kidney volume mediates effect of body size and body composition on renal functional response. (a) Example model, (b) Weight, (c) Body mass index, and (d) Body surface area. Each figure represents 3 regression equations as follows: (i) Independent variable-dependent variable, β1 is the effect estimate (with 95% confidence interval); (ii) Independent variable-mediator, with β2 as the effect estimate; and (iii) independent variable and mediator-dependent variable, with β3 as the effect estimate of the mediator and β4 as the effect estimate of the independent variable. The direct effect of the independent variable is given as β4, whereas the indirect (mediated) effect is β2 × β3 or also β4−β1.

In a *post hoc* analysis, we studied the mediation of the effects of weight, BMI, and BSA on RFR through kidney volume. An outline of the process is shown in Figure 3a. In the regression of weight on RFR, the estimate was 0.1 (*P* = 0.02). However, as can be seen in Figure 3, this effect was mediated through kidney volume. We calculated the indirect (i.e., mediated) effect to 0.08 with a 95% confidence interval after bootstrapping with 5000 simulations to 0.0 to 0.18 (*P* = 0.04). Similar results were seen for BMI and BSA, whereas for BSA the original regression of BSA on RFR was not significant.

## Discussion

To our knowledge, this is the first study to examine the association between birth weight and RFR. It is also the first large scale study using plasma clearance of iohexol to measure RFR. After a protein meal consisting of a protein shake, we found a significant change in GFR. This change was positive in 87% of the participants and yielded RFR >5 ml/min in 67%. In contrast to our hypothesis, we found no association between birth weight or any other birth-related variables and RFR. We could not find any significant sex difference in RFR; however, there may be different underlying mechanisms. In agreement with our hypothesis, larger kidneys were associated with higher RFR.

The lack of association between birth weight or any of the other birth-related variables and RFR, in our study, could have several explanations. Although this could mean that single nephron hyperfiltration is not a feature of the association of birth weight with adult kidney function, this is unlikely, because other studies have provided evidence of such a mechanism.^22^^,^^23^ One explanation could be that the long-term single nephron hyperfiltration in LBW individuals increases the maximum single nephron hyperfiltration attainable, possibly as a result of glomerular hypertrophy. In a study of kidney donors, predonation sGFR did only predict short-term kidney function.^24^ In the long-term, the mGFR of the remaining kidney was clearly higher than half the predonation sGFR (predonation sGFR 126 ml/min, 5 year postdonation random GFR 78 ml/min). Considering that the number of nephrons cannot increase, this suggests the single nephron hyperfiltration increased more than the maximum level of the stress test. The magnitude of RFR in our cohort was lower than those in many other studies in which increases of more than 25 to 40 ml/min is often seen.^2^^,^^5^^,^^25^ Part of this difference might be due to the attributes of the test itself; we tested the mean change of GFR for 4 hours. Other tests using inulin or creatinine clearance have often shown 30-minute intervals, with increases only in 1 or 2 30-minute periods in the 4 hours following the protein load.^2^^,^^5^^,^^25^ In addition, this study was originally designed to compare the mGFR in healthy adults of the 2 birth weight groups without including sex stratification. It might therefore have been underpowered to find a small difference in RFR, especially in the case of a sex difference in the effect of birth weight on RFR. However, a *post hoc* power calculation showed that the study had enough power to show a clinically significant difference in RFR. Age and the exclusion factors of eGFR <60 ml/min per 1.73 m^2^ and hypertension excluded ex-premature individuals with more advanced kidney involvement, thereby falsely giving a milder picture of the renal consequences of LBW.

RFR was higher in individuals with larger kidneys. Although RFR is linked to functional renal mass,^3^ the association between RFR and kidney volume has previously only been investigated in the setting of adult polycystic kidney disease where large kidneys suggest reduced kidney function.^9^ Nephron number is associated with kidney weight^26^ but the association may be complicated by renal hypertrophy, and in a previous study the authors warn against using renal size as a marker of nephron numbers.^27^ Nevertheless, we have previously shown a positive association between kidney volume and GFR in the same cohort.^16^ In the absence of hyperfiltration, this would suggest at least some association between kidney volume and nephron number. We then calculated the kidney function per kidney volume, which we termed the kidney utilization ratio. Assuming a positive association of kidney volume with nephron number, the kidney utilization ratio could act as a crude estimation of single nephron hyperfiltration. We found that this was highly significantly negatively associated with RFR, with no significant difference between men and women. There was also no difference whether RFR was measured as delta GFR, percentage of baseline, or if corrected for BSA or not. Our interpretation is that this association is compatible with the assumption that RFR is reduced when single nephron hyperfiltration is already taking place. The optimal GFR is not known but depends on a person’s age.^28^, ^29^, ^30^ A higher GFR is not necessarily optimal and has been associated with steeper long-term GFR decline in diabetes and the general nondiabetic population.^31^ In a recent study it was found that middle-aged healthy men had a higher bGFR but a steeper and nonlinear GFR decline compared with healthy women.^32^ Uncovering hyperfiltration within the normal GFR range may be difficult;^31^^,^^33^ however, it should be investigated whether the kidney utilization ratio could be a useful tool.

In previous studies, high body weight has been associated with a high resting GFR and hence a lower RFR,^34^^,^^35^ whereas others have found higher RFR in the metabolic syndrome despite having a higher resting GFR.^36^ We found a positive correlation between weight and both bGFR and sGFR (*r* = 0.63 and *r* = 0.68 for baseline and stimulated, respectively; both *P* < 0.001), thereby giving a higher RFR with higher weight. However, in a sensitivity analysis, the effects of body weight on RFR were different in analysis using RFR in percentage or RFR per BSA. In the same sensitivity analysis, we found only minor differences for kidney volume in the unadjusted model only, whereas GFR per kidney volume and GFR per body weight were virtually unchanged. This argues that there are underlying associations between body composition and RFR that are not fully captured in the main analysis. Part of this could be the association between weight and kidney volume (*r* = 0.64, *P* < 0.001) and the previously shown association between kidney volume and GFR.^16^ Indeed, in mediation analysis, we found that the effect of weight on RFR could be mediated through kidney volume. The effect of GFR per kidney volume was similar for both men and women, whereas GFR per body weight was stronger in men. Other possible sex differences were in the association of blood pressure with RFR. In the sex-stratified analysis, there was a negative association between blood pressure and RFR in female participants only. However, because there was only a trend for the sex interaction in the main analysis (*P* = 0.1 and *P* = 0.06 for systolic and diastolic blood pressure, respectively), this must be interpreted with caution.

The strength of this study is the relatively large sample size concerning RFR research. Most previous studies on RFR include less than 50 participants.^1^ Our mGFR method is well established and comparable to the gold-standard method using inulin clearance.^37^ Our population was presumably healthy and of an age group where changes in kidney function are becoming clinically relevant. We had extensive data on almost all participants, including birth-related data from a compulsory registry. The most important weaknesses of this study were that the method for measuring RFR required 2 separate test days. Because we had only 1 measurement per participant of bGFR it is possible that our results were influenced by day-to-day variations; however, this is generally quite low.^38^ We used the ellipsoid formula for calculating kidney volume, which is the easier method but less accurate compared with kidney volume measured by disk summation.^39^ This method was nevertheless used for the whole cohort, and we believe that this would reduce most inaccuracies caused by the method because of the size of the cohort. Furthermore, the cohort size was determined from a power calculation for the difference in mGFR and this was probably not transferable to this study. The lack of dietary restrictions except fasting on the morning of the baseline test may have influenced the results.

In mainly healthy middle-aged individuals, we did not find any association between RFR and birth weight or any other birth-related variables. Despite this, we found evidence that larger kidneys associate with higher RFR. Although most methods for measuring RFR are cumbersome, the kidney utilization ratio calculated as the ratio between kidney function and kidney volume shows a good correlation with RFR, and future studies should investigate this further. Our results for RFR and kidney volume should be tested using other methods of measuring GFR in the estimation of RFR. We would also stress the importance of a larger cohort for sufficient statistical power.

## Disclosure

All the authors declared no competing interests.

## Ethics

The study was approved by the regional ethics committee (REK2017/927). All participants signed a written informed consent before inclusion in the study. The data underlying this article will be shared on reasonable request to the corresponding author.

## Supplementary Materials

Supplementry File (PDF)

**Figure S1.** Distribution of renal functional response by birth weight group and sex.

**Figure S2.** Bland Altman plot showing association between stimulated and baseline GFR.

**Table S1.** Sex-stratified association between renal functional response and various variables using regression.

**Table S2.** Sensitivity analysis.
