# Supplementary material for: Renal Functional Response-Association With Birth Weight and Kidney Volume
Source: Kidney Int Rep. 2023 Feb 20;8(5):1034–42. doi: 10.1016/j.ekir.2023.02.1079 (PMC10166784; doi:10.1016/j.ekir.2023.02.1079)
Supplement: Supplementry File (PDF) [file mmc1.pdf]

Supplementary material

Content of supplementary materials:

Supplementary table 1 – Sex- stratified association between Renal Functional Response and various variables using regression

Supplementary table 2 – Sensitivity analysis

Supplementary figure 1 – Distribution of Renal Functional Response by birth weight group and sex

Supplementary figure 2 - Bland Altman plot showing association between stimulated and baseline GFR

Supplementary table 1 - Sex-stratified association between Renal Functional Response and various variables using regression												
	Male						Female					
	Modell 0		Modell 1		Modell 2		Modell 0		Modell 1		Modell 2	
	Estimate	p-value	Estimate	p-value	Estimate	p-value	Estimate	p-value	Estimate	p-value	Estimate	p-value
Low birth weight	1.12	0.6	-0.57	0.8	-0.14	0.9	0.41	0.8	2	0.3	1.25	0.5
Birthweight	-0.73	0.5	0.08	0.9	-0.04	0.9	-0.06	0.9	-0.89	0.4	-0.44	0.7
Birthweight per gestational age	-0.24	0.8	-0.04	0.9	0.11	0.9	0.74	0.4	0.28	0.8	0.73	0.5
Gestational age	-0.71	0.6	0.05	0.9	-0.07	0.9	-0.19	0.8	-0.81	0.4	-0.62	0.5
Preterm	1.64	0.5	0.01	0.9	0.51	0.8	0.57	0.8	1.88	0.3	1.66	0.4
Age	-1.24	0.3	-1.97	0.09	-1.09	0.3	-1.11	0.2	-1.27	0.2	-1.73	0.07
Weight	2.5	0.03	3.56	0.004	4.77	<0.00	1.15	0.2	0.94	0.3	2	0.09
Height	-0.86	0.5	-1.79	0.1	-1.66	0.1	1.38	0.1	1.18	0.2	1.53	0.1
Body Mass Index	2.94	0.01	6.15	0.8	10.14	0.6	0.84	0.4	24.05	0.1	18.18	0.3
Body Surface Area	1.76	0.1	0.27	0.9	18.01	0.3	1.36	0.1	1.1	0.9	-1.45	0.9
Systolic blood pressure	0.24	0.8	-1.5	0.2	-1.1	0.4	-2.04	0.02	-2.83	0.008	-2.59	0.02
Diastolic blood pressure	0.57	0.6	-0.09	0.9	0.34	0.8	-2.12	0.02	-2.36	0.02	-2.09	0.06
Kidney volume	2.01	0.08	1.06	0.5	3.24	0.02	2.39	0.009	2.05	0.1	4.59	0.001
Measured GFR	-2.06	0.08	-3.44	0.005	-3.44	0.005	0.27	0.8	-1.82	0.1	-1.82	0.1
GFR per kidney volume	-4.27	<0.00	-3.71	<0.00	-2.55	0.03	-2.72	0.002	-2.94	0.001	-2.82	0.005
GFR per kg body weight	-4.14	<0.00	-3.83	0.003	-2.98	0.4	-1.1	0.2	-1.52	0.2	0.85	0.8

All estimates are given per SD, except the dichotome variables birth weight group and preterm. Model 0 is unadjusted, Model 1 is adjusted for age, weight and height, and model 2 is adjusted for age, weight, height and baseline GFR.

Supplementary table 2. Sensitivity analysis						
	Model 0		Model 1		Model 2	
	Estimate	P-value	Estimate	P-value	Estimate	P-value
Low birth weight (RFR= delta GFR)	0.10	0.6	0.13	0.5	0.02	0.9
Low birth weight (RFR = %)	0.19	0.3	0.21	0.3	0.06	0.8
Low birth weight (RFR = BSA corrected)	0.11	0.6	0.13	0.5	0.03	0.9
Birthweight (RFR= delta GFR)	-0.05	0.6	-0.07	0.5	-0.0004	0.9
Birthweight (RFR = %)	-0.1	0.3	-0.12	0.3	-0.03	0.8
Birthweight (RFR = BSA corrected)	-0.06	0.6	-0.07	0.5	-0.01	0.9
Birth weight per gestational age (RFR= delta	0.04	0.7	0.01	0.9	0.07	0.5
Birth weight per gestational age (RFR = %)	-0.01	0.9	-0.03	0.8	0.06	0.5
Birth weight per gestational age (RFR = BSA	0.02	0.8	0.01	0.9	0.06	0.5
Gestational age (RFR= delta GFR)	-0.06	0.6	-0.06	0.6	-0.02	0.8
Gestational age (RFR = %)	-0.08	0.4	-0.09	0.4	-0.03	0.7
Gestational age (RFR = BSA corrected)	-0.05	0.6	-0.05	0.6	-0.02	0.9
Preterm (RFR= delta GFR)	0.14	0.5	0.13	0.5	0.09	0.7
Preterm (RFR = %)	0.16	0.4	0.17	0.4	0.11	0.6
Preterm (RFR = BSA corrected)	0.11	0.6	0.12	0.6	0.08	0.7
Age (RFR= delta GFR)	-0.16	0.1	-0.2	0.04	-0.22	0.02
Age (RFR = %)	-0.16	0.1	-0.18	0.07	-0.22	0.02
Age (RFR = BSA corrected)	-0.19	0.05	-0.21	0.04	-0.23	0.02
Weight (RFR= delta GFR)	0.22	0.02	0.3	0.007	0.49	<0.001
Weight (RFR = %)	0.08	0.4	0.17	0.1	0.44	<0.001
Weight (RFR = BSA corrected)	0.09	0.4	0.18	0.1	0.36	0.005
Height (RFR= delta GFR)	0.03	0.7	-0.05	0.7	0.01	0.9
Height (RFR = %)	-0.05	0.6	-0.03	0.8	0.05	0.7
Height (RFR = BSA corrected)	-0.04	0.7	-0.06	0.7	0	0.9
Body Mass Index (RFR= delta GFR)	0.23	0.02	-0.55	0.6	-0.44	0.7
Body Mass Index (RFR = %)	0.11	0.3	-0.38	0.7	-0.22	0.8
Body Mass Index (RFR = BSA corrected)	0.12	0.2	-0.24	0.8	-0.13	0.9
Body Surface Area (RFR= delta GFR)	0.18	0.07	-1.49	0.4	-0.68	0.7
Body Surface Area (RFR = %)	0.04	0.7	-1.27	0.4	-0.06	0.9
Body Surface Area (RFR = BSA corrected)	0.05	0.6	-0.94	0.6	-0.15	0.9
Systolic blood pressure (RFR= delta GFR)	-0.14	0.2	-0.31	0.006	-0.26	0.02
Systolic blood pressure (RFR = %)	-0.22	0.02	-0.35	0.002	-0.27	0.01
Systolic blood pressure (RFR = BSA	-0.2	0.04	-0.32	0.005	-0.27	0.02
Diastolic blood pressure (RFR= delta GFR)	-0.11	0.3	-0.2	0.07	-0.13	0.2
Diastolic blood pressure (RFR = %)	-0.2	0.04	-0.24	0.03	-0.14	0.2
Diastolic blood pressure (RFR = BSA	-0.17	0.08	-0.2	0.07	-0.14	0.2
Kidney volume (RFR= delta GFR)	0.25	0.009	0.33	0.02	0.69	<0.001
Kidney volume (RFR = %)	0.1	0.3	0.21	0.1	0.65	<0.001

Supplementary table 2. Sensitivity analysis						
	Model 0		Model 1		Model 2	
	Estimate	P-value	Estimate	P-value	Estimate	P-value
Kidney volume (RFR = BSA corrected)	0.18	0.07	0.33	0.02	0.69	<0.001
Measured GFR (RFR= delta GFR)	-0.08	0.5	-0.38	0.003	-0.38	0.003
Measured GFR (RFR = %)	-0.26	0.007	-0.55	<0.001	-0.55	<0.001
Measured GFR (RFR = BSA corrected)	-0.15	0.1	-0.36	0.006	-0.36	0.006
GFR per kidney volume (RFR= delta GFR)	-0.45	<0.001	-0.47	<0.001	-0.43	<0.001
GFR per kidney volume (RFR = %)	-0.48	<0.001	-0.5	<0.001	-0.4	<0.001
GFR per kidney volume (RFR = BSA	-0.45	<0.001	-0.47	<0.001	-0.43	<0.001
GFR per kg body weight (RFR= delta GFR)	-0.31	0.001	-0.29	0.009	0.15	0.6
GFR per kg body weight (RFR = %)	-0.37	<0.001	-0.45	<0.001	-0.01	0.9
GFR per kg body weight (RFR = BSA	-0.25	0.009	-0.29	0.01	0.02	0.9

Comparison of regression models using the z-score of three different RFR models as dependent variable: RFR as delta GFR, RFR as percentage change from baseline, and RFR as delta GFR using BSA corrected GFR. Estimate is per SD of main variable, except for the dichotome variables Low birth weight, and Preterm.

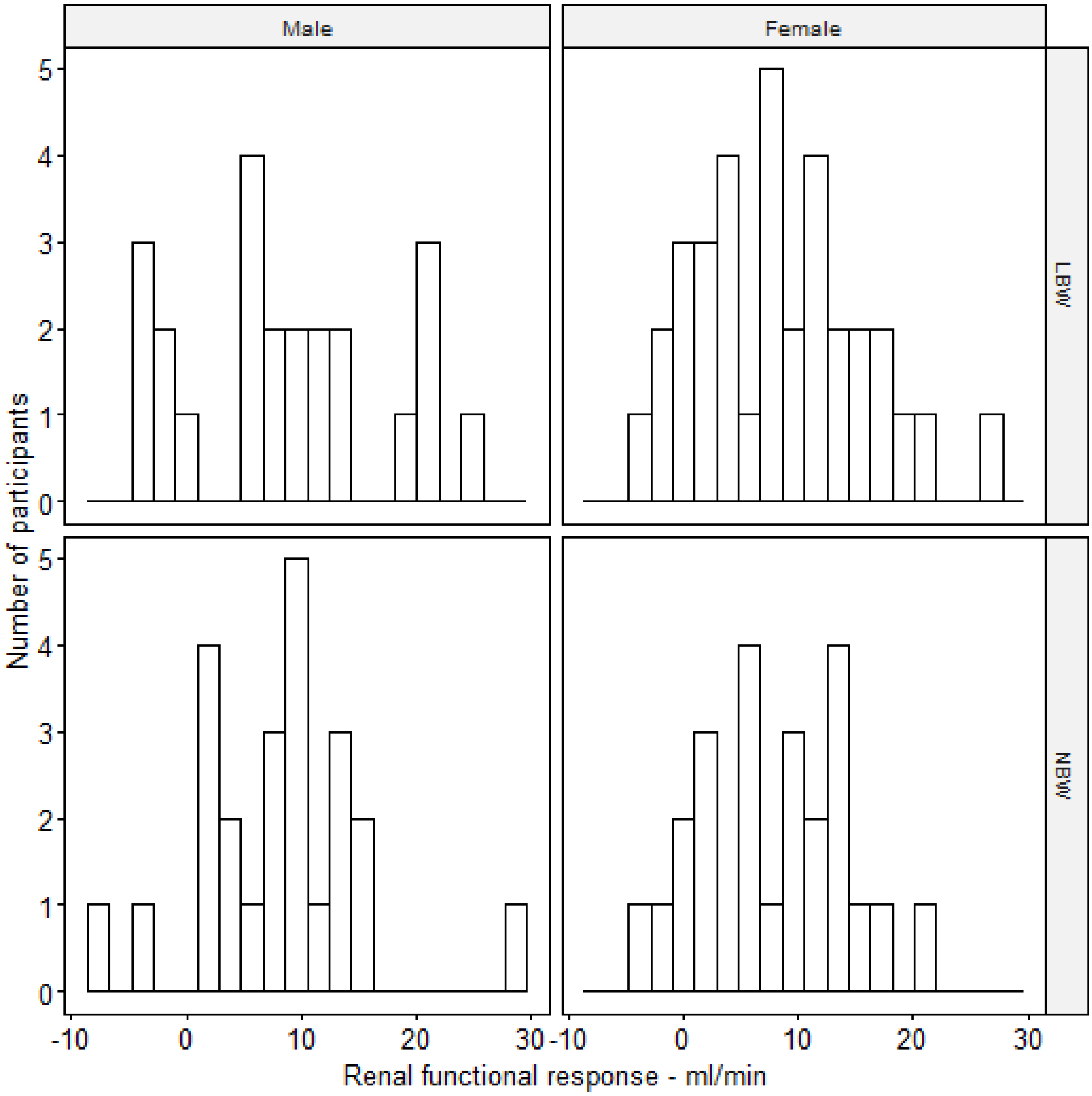

Supplementary figure 1:
Title: Distribution of Renal functional response by birth weight group and sex
Legend:

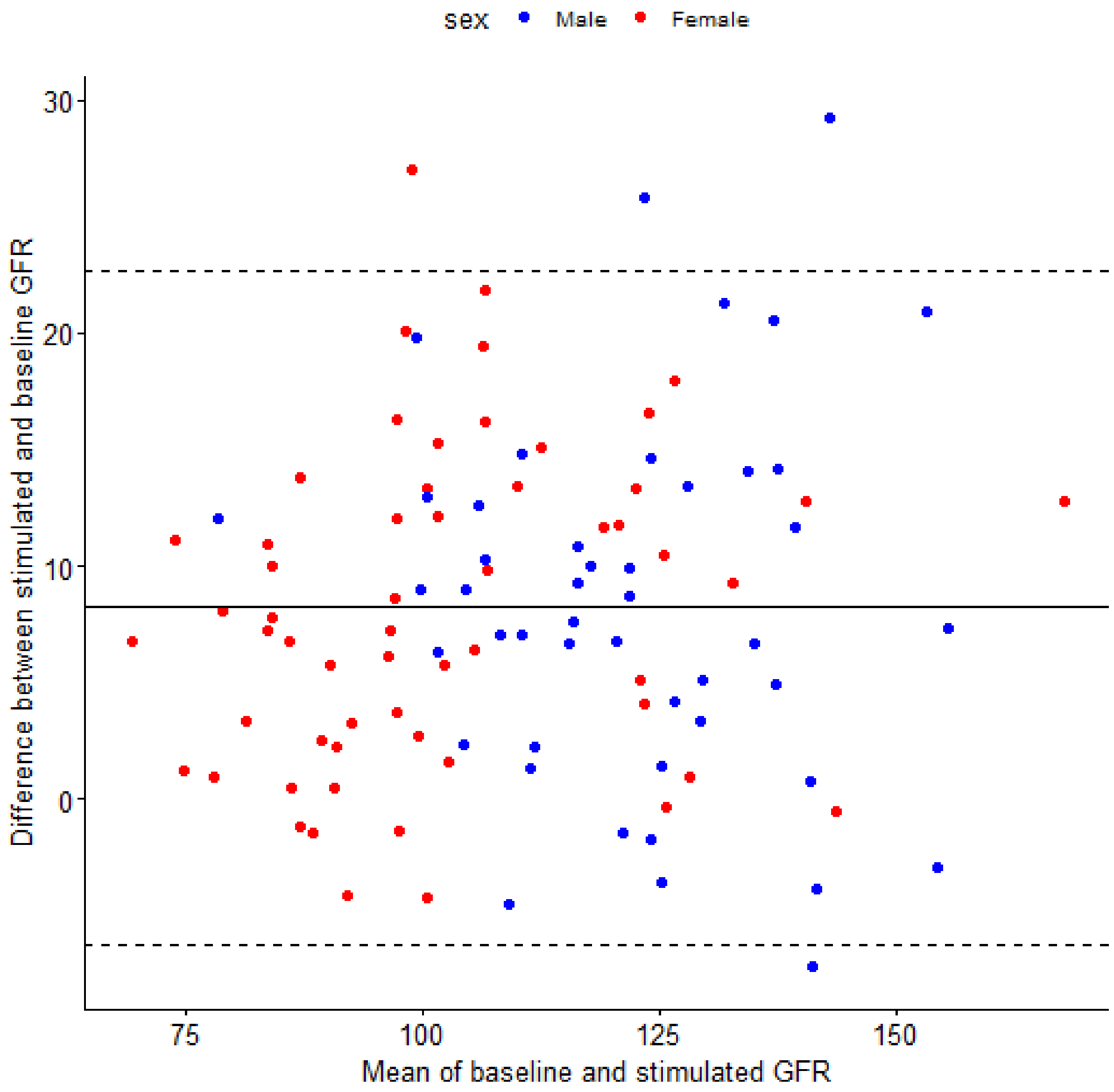

Supplementary figure 2:

Title: Bland Altman plot showing association between stimulated and baseline GFR

Legend:

All participants represented by a dot, colored red for female, and blue for male. Solid horizontal line represents the mean difference between stimulated and baseline GFR, while the dashed lines represents the 95% confidence interval.
